# Identification of novel host-oriented targets for Human Immunodeficiency Virus type 1 using Random Homozygous Gene Perturbation

**DOI:** 10.1186/1743-422X-6-154

**Published:** 2009-09-29

**Authors:** Hanwen Mao, Hanson Chen, Zena Fesseha, Shaojing Chang, Huong Ung-Medoff, Jessica Van Dyke, Manu Kohli, Wu-Bo Li, Michael Goldblatt, Michael S Kinch

**Affiliations:** 1 Functional Genetics, Inc. 708 Quince Orchard Road, Gaithersburg, MD 20878, USA

## Abstract

**Background:**

Human Immunodeficiency Virus (HIV) is a global threat to public health. Current therapies that directly target the virus often are rendered ineffective due to the emergence of drug-resistant viral variants. An emerging concept to combat drug resistance is the idea of targeting host mechanisms that are essential for the propagation of the virus, but have a minimal cellular effect.

**Results:**

Herein, using Random Homozygous Gene Perturbation (RHGP), we have identified cellular targets that allow human MT4 cells to survive otherwise lethal infection by a wild type HIV-1_NL4-3_. These gene targets were validated by the reversibility of the RHGP technology, which confirmed that the RHGP itself was responsible for the resistance to HIV-1 infection. We further confirmed by siRNA knockdowns that the RHGP-identified cellular pathways are responsible for resistance to infection by either CXCR4 or CCR5 tropic HIV-1 variants. We also demonstrated that cell clones with these gene targets disrupted by RHGP were not permissible to the replication of a drug resistant HIV-1 mutant.

**Conclusion:**

These studies demonstrate the power of RHGP to identify novel host targets that are essential for the viral life cycle but which can be safely perturbed without overt cytotoxicity. These findings suggest opportunities for the future development of host-oriented therapeutics with the broad spectrum potential for safe and effective inhibition of HIV infection.

## Background

Therapy-resistant HIV-1 strains are relentlessly emerging as a result of the error-prone HIV viral reverse transcriptase, robust viral replication and incomplete patient compliance. In some regions, viruses that are resistant to drug cocktail therapy or HAART (Highly Active Antiretroviral Therapy) were isolated from nearly 20% of AIDS patients evaluated [1,2]. Such findings increase the urgency to identify new paradigms for the treatment of HIV/AIDS, especially mechanisms of action that are relatively insensitive to the development of resistance.

It is well established that interplay between the viruses and host cells determines the outcome of viral pathogenesis, ranging from the elimination of viruses to latent or lethal infections. HIV-1 is known to interact with host cellular proteins to aid their replication and evade immune attack. One example involves individuals who carry a defective cell surface receptor (CCR5) and have been shown to be resistant to HIV-1 infection [3,4]. Similar interactions have been reported to encompass nearly every step of HIV-1 life cycle: from viral entry [5] to viral budding and release [6]. Such findings suggest that increased understanding of the interaction of HIV-1 with host protein could improve therapeutic and prevention strategies to combat HIV/AIDS.

In light of the understood importance of host factors in HIV-1 infection, increasing investigation has begun to consider host targets for antiviral therapy. Specifically, host targets that are essential for HIV-1 replication, but not for the host cell itself, could provide a new modality of treatment. It is further postulated that certain host targets might not place direct selective pressure on the pathogen and thus minimize the acquisition of drug resistance. Host-directed therapeutics has begun to be successfully deployed against HIV/AIDS, including treatments that target the CD4 viral receptor and associated co-receptors [7,8]. Indeed, some of the newest approved and most promising experimental therapeutic options include small molecules or biologics that target these host proteins.

Not all host molecules are suitable as therapeutic targets as many serve essential functions for the growth, function or survival of host cells. However, it is increasingly understood that viruses often circumvent the expression or function of some host proteins (in a process known as "hijacking") and this may provide an opportunity to target host molecules that are inappropriately expressed or functionally altered in HIV-infected cells. To identify such targets, our laboratory has employed a novel technology, Random Homozygous Gene Perturbation (RHGP), to select for targets that are essential for HIV infection but which are not necessary for the growth, survival or function of non-infected cells. RHGP was designed to allow the investigator to up- or down-regulate any gene in a eukaryotic cell, independent of any prior knowledge or annotation of that gene [9]. In this manner, RHGP provides an un-biased approach to identify any target, whether up- or down-regulated, which is responsible for a desired phenotype. As one example, our laboratory has successfully used RHGP to identify and validate target genes that allow host cells to survive an otherwise lethal infection with Influenza A virus[10]. Of 110 targets identified by this genome wide screen technology, most (106 of 110) had not been described previously or linked with influenza infection. In addition, we ascribed novel functions to previously unknown genes and orfs (open reading frames).

Herein, we apply RHGP and identify a set of host-oriented targets that allow host cells to resist lethal HIV infection. These novel targets include both known genes and non-annotated ESTs (Expression Sequence Tags), whose functions have not been assigned. We validated these genes using unique properties of the RHGP technology as well as independent genetic targeting approaches such as siRNA. This investigation provides increased understanding of the interplay between host targets and HIV and could provide potential therapeutic targets to combat HIV/AIDS.

## Methods

### Cell lines and Viruses

The following cell lines, viruses and proviral molecular clones were obtained through the AIDS Research and Reference Reagent Program, Division of AIDS, NIAID, NIH: MT4 cells from Dr. Douglas Richman, PM1 cells from Dr. Marvin Reitz [11], TZM-bl cells from Dr. John C. Kappes, Dr. Xiaoyun Wu and Tranzyme Inc. [12], pNL4-3 from Dr. Malcolm Martin[13], HIV-1_ME1 _from Dr. Phalguni Gupta [14] and Protease-resistant HIV-1 (L10R/M46I/L63P/V82T/I84V) from Dr. Emilio Emini[15]. MT4 and PM1 cells were grown in RPMI 1640 medium containing 10% heat-inactivated FBS (HyClone, Long, UT) supplemented with 2 mM glutamine (Invitrogen), 2-mercaptoethanol (50 μM), 100 μg/ml streptomycin (Invitrogen). TZM-bl cells were cultured in DMEM containing 10% FBS and 100 μg/ml streptomycin. HIV-1_NL4-3 _was made from HEK293 after transfection with the proviral DNA followed by amplification in MT4 cells.

### HIV-1 Infection and Measurements of Viral Production

MT4 or PM1 cells were infected with HIV-1 at a multiplicity of infection (MOI) of 0.001 by low speed centrifugation (1,200 g) for 1 hr. The use of a relatively low MOI helped us to identify host factors whose anti-viral effects may not be robust or directly acting on virus replication and which will be more likely discovered after multiple cycles of viral replication. Supernatants collected post infection were then transferred to the TZM-bl indicator cell line for determination of infectious viral particles. Relative Luminescence Unit (RLU) was obtained on TZM-bl cells after they were treated with Bright-Glo Luciferase Assay System (Promega) 3 days post infection (dpi). Amounts of p24 in the collected supernatants were measured using HIV-1 p24 ELISA kit (Xpressbio, Thurmont, MD, USA) following the manufacturer's instructions.

### Description of RHGP technology

RHGP utilizes a unique genetic element, known as a gene search vector (GSV), which is based on a retrovirus or lentivirus backbone. The GSV was designed to interrogate the entire genome and identify targets without any prior knowledge and that allow host cells to resist or survive lethal HIV-1 infection.

**Figure 1:**
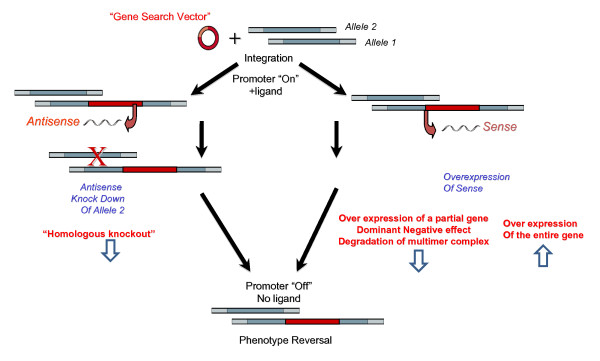
**An overview of potential outcomes of GSV integration into the genome**. The left panels demonstrate how integration of the Gene Search Vector in an "antisense orientation" would disrupt Allele 1 and then facilitate overexpression of an antisense to disrupt the second allele in the presence of inducer (RSL1). On the other hand, the right panels demonstrate how integration in a sense orientation would facilitate overexpression of the target gene (or domains thereof). Note that the phenotype is tightly regulated by the RSL1-inducible promoter, thus allowing the investigation to reverse the phenotype by turning the GSV vector "off" when cells are cultured in the absence of RSL1.

As demonstrated previously (reference [10]) and modified in Figure 1, our experimental strategy makes use of integration of the GSV at a single site in the genome, where it regulates expression of the target gene via an inducible promoter. The GSV could integrate in either a sense or an antisense orientation. In the antisense configuration, the integration event itself inactivates one allele and facilitates expression of an antisense construct, which further knocks down expression of genes encoded on the other allele (Figure 1, left panels). In this way, RHGP generates homozygous perturbation of both gene copies in diploid cells. When GSV integrates in the sense orientation, RHGP facilitates over-expression of the target gene (Figure 1, right panels). This outcome could lead to over-expression of an entire gene when insertion is upstream of the start codon or expression of specific domains initiated from a downstream endogenous start codon when integration occurs within a gene. This newly truncated protein could produce a dominant-negative inhibitor. In the case when the wild type protein has a tendency to form a dimer or multimer, the mutant partner thus triggers rapid degradation of the complex due to misfolded aggregates they form into. As such, RHGP allows us to sample the entire cell genome to identify different types of events that render host cells to resist or survive HIV-1 infection. The transcript production is under control of a ligand inducible promoter that carried in the GSV and viral resistance is derived in the presence of the ligand (promoter "On"). The causal relationship between the disturbed genotype and viral resistant phenotype can be confirmed by withdrawal of the ligand (promoter "Off").

### Construction of the MT-4 R1 Cell Lines

RheoSwitch^® ^Mammalian Inducible Expression System was purchased from New England Biolabs (NEB). Plasmid pNEB-R1 encoding the transactivator R1 was first linearized using the restriction enzyme ScaI (NEB). MT4 cells were then transfected with the linearized pNEB-R1 by electroporation using Eppendorf Multiporator (Eppendorf, AG 22331, Hamburg, Germany) under conditions of 360 v (voltage) and 100 μs (time constant). The transfected MT4 cells were selected using G-418 (400 μg/mL) and G-418 resistant cells were cloned by serial limited dilutions. After expansion, clones were examined at least twice for luminescence (relative luminescence units (RLUs)) after transfection with an R1-responsive luciferase reporter gene (pGluc, NEB) using the Gaussia Luciferase Assay Kit (NEB). We determined the RSL1 induction folds of luminescence from these cell clones as: RLUs obtained from samples in the presence of the inducer divided by RLUs from samples without the inducer treatment. The induction fold from these clones ranged from 2-60 folds. A stable clone (#2-14) with the highest induction was chosen to create RHGP libraries.

### Construction of the RHGP Gene Search Vector, pRHGP12-RSN

**Figure 2:**
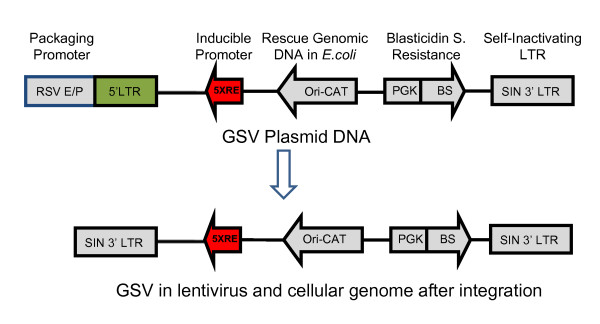
**An overview of the pRHGP12-RSN Gene Search Vector (GSV)**. Unique and important features of the vector are highlighted that facilitate the RHGP-based identification of host-based gene targets contributing to HIV-1 resistance. Note that the promoter used for production of the GSV "Packaging Promoter" is not included into the GSV viral genome and the 5' LTR is replaced with the SIN 3'LTR after the vector integrates into the cellular genome.

The RHGP gene search vector, pRHGP12-RSN, was constructed using the lentivirus-based pLEST vector as a backbone (generously provided by Dr. Stanley Cohen, Stanford) [16]. This vector was constructed with RheoSwitch Mammalian Inducible System (NEB) (Figure 2). The Rheoswitch system contains five copies of the GAL4 response element (5 × RE) upstream of a TATA box that results in high induction of transcription with low basal expression in the presence of RSL1 ligand. To construct the vector, the DNA sequence of Neo^R ^-TRE-CMV in pLEST was first replaced with a RheoSwitch (RS) inducible Expression cassette containing Ori-CAT-RS in an orientation inverted to that of 5'LTR. The selection marker and reporter cassette containing the Blasticidin (BS) resistant gene and an EGFP gene controlled by a PGK promoter was inserted in the NheI site in an orientation opposite to the RS expression cassette.

### Production of Lentivirus Carrying GSV and Construction of RHGP Library

RHGP lentiviruses were produced using ViroPower Expression System (Invitrogen). HEK293FT cells were plated in 10 cm plates at 10^6 ^cells per plate. After 24 h incubation, the cells were transfected with 3 μg RHGP12-RSN and 9 μg ViroPower Packaging Mix using Lipofectimine 2000 (Invitrogen). The medium was changed after 5 h incubation. After 48 h, viruses in the culture medium were filtrated through a 0.45 μm filter and titrated according to the manufacturer's instruction.

To construct the RHGP library, MT4-R1 cells were transduced with RHGP viruses in the presence of polybrene (6 μg/mL) by low speed centrifugation (1,200 g) for 1 h. To minimize the potential for multiple insertions within a single cell, a low MOI (0.1) was employed during the library creation to minimize the likelihood that cells might be transduced by more than one different GSV. GSV integrated cells were selected using GBL medium (complete RPMI 1640 medium containing G418 (400 ug/ml), Blasticidin (4 ug/ml) and RSL1 ligand (0.5 uM)).

### Selection of RHGP Cell Clones That Survived from HIV-1 Challenge and Confirmation via Reversibility of Viral Resistance

After challenge with HIV-1_NL4-3_, the MT4-R1 RHGP library was cultured in the same GBL medium described above. The individual surviving clones were established by serial limited dilutions and continuously expanded in GBL medium. Cell clones were further challenged with HIV-1_NL4-3 _to confirm their resistance.

To verify reversibility of RHGP induced events, the viral resistant MT4 cell clones were cultured in the GBL medium or GBL medium without RSL1 separately for at least 3 days before HIV-1 re-challenge. Viral production (infectivity and p24) in supernatants were examined as described above.

### Identification of Candidate Genes from Virus Resistant Clones

The RHGP gene search vector was designed to efficiently discover target genes and determine the orientation (sense or antisense) of an integration event. The gene search vector contains an Ori-CAT reporter gene (Figure 2), which can be rescued by restriction enzyme-based genomic DNA cloning as described before[10]. Briefly, cellular genomic DNA from each cell clone was extracted from 10^6 ^cells. Purified genomic DNA was then digested with BamHI or XbaI and self-ligated overnight using T4 ligase (Invitrogen). The ligated DNA was electroporated into DH10B ElectroMax competent cells (Invitrogen). After overnight growth, multiple colonies were isolated for plasmid DNA preparation and restriction enzyme digestion. The plasmid DNA was further used to identify the target genes by DNA sequencing and genome mapping. The resulting genomic DNA sequences flanking the RHGP vector insertion sites were subjected to genome mapping against the human genome using the UCSC Genome Browser .

### Validation of Identified Host Target Genes with siRNA Knockdown Assay

Human duplex siRNA (siGNOME SMARTpool) for RHGP identified genes were prepared as recommended by the manufacturer (Dharmacon). The siRNA Rab6A and HIV-1 Tat were employed as positive controls [17]. Non-targeting siRNA (siCONTROL1) was used as a negative control. MT4 or PM1 cells were cultured in fresh complete RPMI 1640 medium overnight. The log-phase growing cells were transfected with 1.2 uM of siRNA by electroporation, according to the manufacturer's instruction (Eppendorf). The voltage and time constant for elctroporation were 360 *v*, 100 μ*s *and 200 *v*, 200 μ*s *for MT4 and PM1 cells, respectively. The cells were infected with HIV-1 variants 24 h post transfection. Culture media were refreshed everyday and the cell viabilities were examined daily by trypan blue dye exclusion assay. Viral production (infectivity and p24) in supernatants were examined as described above.

### Western Blot Analysis

The cell pellets were washed with PBS, resuspended with lysis buffer (25 mM Tris-HCl, pH 7.6, 150 mM Nacl. 1% NP-40. 1% Sodium Deoxycholate, 0.1% SDS), and disrupted with pulse sonication. After centrifuge at ≅14000 × g for 15 minutes, the supernatant was dialyzed against PBS, and concentrated. Equal amounts of protein samples (250 μg) were loaded onto 4%-12% stacking SDS-PAGE with Dithiothreitol (DTT, 200 mM) before electrophoresis analysis. The sieved proteins were transferred on to PVDF membranes, blocked with PBS containing 5% dry non-fat milk, and blotted with 1:50 dilution of anti-Robo1 (A301-265A, Bethyl Laboratories) as primary antibody and 1: 2000 as secondary antibody HRP in PBS containing 5% dry non-fat milk, 0.1% Tween-20 and the ECL Chemiluminescence (Amersham/Pharmacia Biotech) was used to detect signals. The loading amounts controls were probed using anti-HSP (heat shock protein 90) (abcam, Cambridge, MA) and anti-GAPDH (Santa Cruz Biotechnology, Santa Cruz, CA).

## Results

### Construction of the RHGP library of CD4^+ ^T cell line MT4

To identify novel targets that render T cells resistant to HIV infection, we utilized the human MT4 cell model, which provided an HIV-1 permissive, CD4 positive T lymphocyte cell line. The use of a natural target line for a wild-type strain of HIV-1 provided a model to identify targets that are physiologically relevant to the HIV life cycle. In addition, MT4 cells were selected for these studies, in part, after confirming that this model was highly sensitive to HIV-1 infection. Specifically, challenge of MT4 cells with HIV_NL4-3_, at a relatively low initial MOI (0.001), was sufficient to eliminate MT4 cells in the absence of RHGP-mediated gene perturbation.

**Figure 3:**
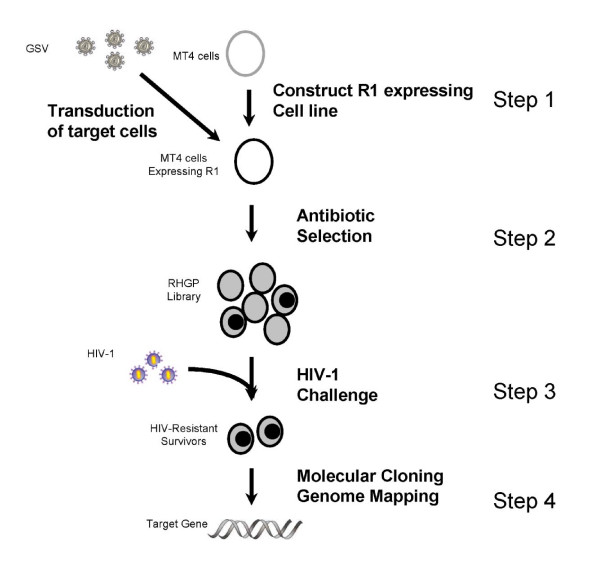
**Schematic overview of the experimental strategy to identify host genes involved in HIV-1 infection using RHGP**. MT4 cell lines expressing the transactivator R1 were first constructed. Following transduction with the RHGP vector antibiotic selection was used to establish an "RHGP library" of gene perturbations. Next the RHGP library cells were challenged with a lethal infection of HIV-1 virus in the presence of the inducer RSL1. Survivors were cloned and then validated by reversing the RHGP phenotype in the absence of RSL1. The genomic DNA was then isolated from those validated clones and the identity of the target gene, along with the orientation of the GSV integration event ("Sense" or "Antisense") was then determined.

**Figure 4:**
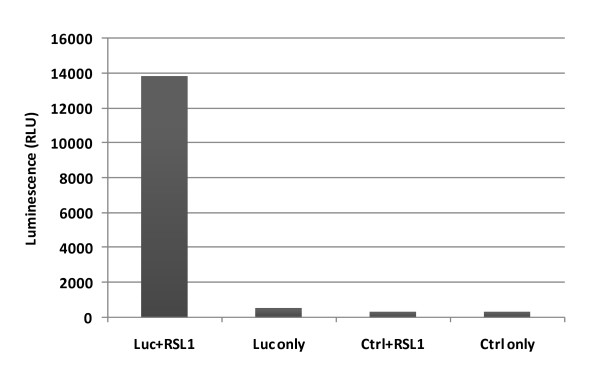
**Activation of a luciferase reporter gene by inducer RSL1 in the MT4-R1 cell line expressing RheoSwitch R1**. Cells were transfected with plasmid DNA encoding a luciferase gene (Luc) or a control plasmid DNA (Ctrl) in the presence or absence of the inducer RSL1. Luminescence was measured 48 h after transfection.

A key feature of the RHGP technology is the ability to validate candidate targets via regulation by an inducible promoter 5xRE (Figure 2). MT4 T cells were first engineered to stably express a transactivator (known as R1), which can activate the built in promoter 5XRE in RHGP to produce transcripts in the presence of the inducer RSL1 (Figure 3, step 1). MT4-R1 cells were thus transfected with an R1-responsive luciferase reporter gene and cultured in the presence or absence of the inducer RSL1. Luminescence readings (RLUs) demonstrated that the resulting MT4-R1 cells generated high and stable levels of luminescence, but only in the presence RSL1 (Figure 4). This result indicated that the activation ability of R1 on the promoter 5xRE is tightly controlled by RSL1. Similar to its parental MT4 cells, we confirmed that these cells retained their susceptibility to HIV-1 infection as complete cell loss was observed after infection of HIV-1_NL4-3_.

We then utilized RHGP to interrogate the genome of human T lymphocytes to identify targets that allow these cells to survive an otherwise lethal infection with HIV-1. To accomplish this, cultures of MT4-R1 cells were transduced with the GSV (pRHGP12; Figure 2), which contains an expression cassette consisting of a constitutive promoter driving a Blasticidin resistance gene. Blasticidin selection allowed us to establish an "RHGP library" of MT4-R1 cells with different genetic perturbations rendered by random GSV integrations (Figure 3, step 2). To maintain stable R1 expression and GSV integration, the MT4-R1 RHGP library was continuously incubated with G418 and Blasticidin. RSL1 was also included in the culture medium to ensure that the activated GSV promoter was able to generate anticipated RHGP effects by producing transcripts. To control for the quality of the library, we confirmed that downstream gene expression from the GSV was induced only upon incubation with RSL1 but not when RSL1 was absent (data not shown). Statistical analyses of gene expression and genome size were implemented to ensure that a sufficient number of GSV integration events would be analyzed to thoroughly evaluate the human genome, both for gain or loss of target expression. Specifically, we calculated that a library of MT4-R1 cells with 10^5 ^GSV integration events would ensure coverage of the human genome[18].

### Isolation of cell clones resistant to HIV-1 infection

The cell library containing the different RHGP perturbation MT4 cells was then challenged with HIV_NL4-3_, infected at an initial MOI of 0.001 (Figure 3, step 3). Analysis of Trypan blue exclusion examination indicated that non-transduced MT4-R1 cells were greater than 99% depleted following HIV-1_NL4-3 _challenge. As indicated above, we also confirmed that the inclusion of RSL1 in non-transduced cells did not alter cell sensitivity to HIV-1 infection. As an additional control, parallel cultures of mock-transduced cells were treated identically and no survivors were observed after 5 days. These controls confirmed that surviving cells arose as a result of the RHGP perturbation and not as an artifact of spontaneous resistance to HIV-1. The small number of surviving cells was cloned and expanded. The resulting clones were then subjected to multiple rounds of challenge to eliminate any susceptible cells. Ultimately, we obtained 25 different cell clones that were insensitive to the lethal HIV-1 challenge.

**Figure 5:**
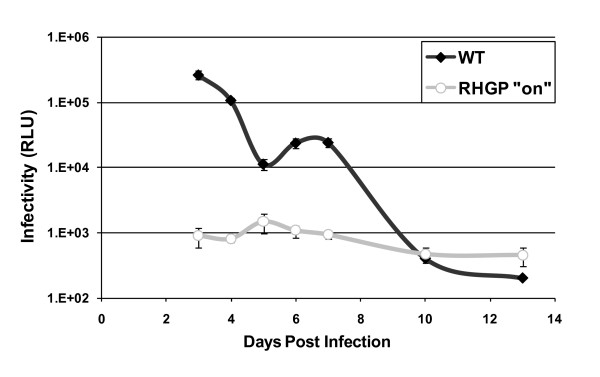
**Loss of viral production from HIV-1 resistant cell clones upon HIV-1 re-challenge**. Production of progeny virus from a representative resistant clone after re-challenge by HIV-1_NL4-3_. Supernatants collected daily starting 3 dpi from the cell cultures were then examined for viral amounts using TZM-bl cells.

Although our results indicated that the RHGP technology prevented HIV-mediated killing of infected cells, we could not exclude that these cells were able to stay alive and yet propagate virus (a less desirable phenotype). We thus asked if the resistant cell clones carrying GSV continued to produce viral particles upon HIV infection. After re-challenge of these resistant cell clones with HIV-1_NL4-3 _in the presence of RSL1, the supernatants were collected at 4 days post infection (dpi) and were transferred to the TZM-bl cells, which provided readout of infectivity. Notably, the RHGP cell clones failed to produce and release progeny virus (See Figure 5 for a representative finding). In contrast, HIV-1 established a productive infection in non-transduced MT4-R1 cells and was ultimately cytotoxic. We confirmed these findings by independently demonstration of diminished p24 levels in the supernatants of RHGP-perturbed clones (data not shown). Thus, we were able to confirm that the RHGP-mediated resistance to HIV killing related directly to elimination of virus propagation.

**Figure 6:**
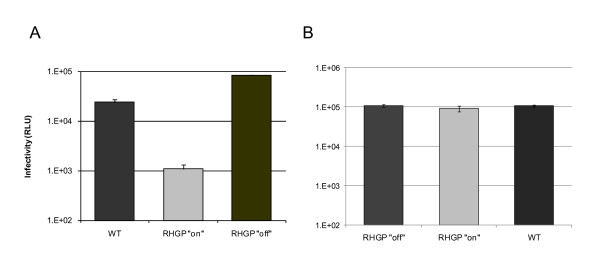
**Validation of cell clones resistant to HIV-1 infection with the reversibility assay**. HIV-resistant RHGP Clone 1-13(**A**) and a naive RHGP Clone H6 (**B**) were challenged with HIV-1_NL4-3 _in the presence (RHGP "on") or absence (RHGP "off") of the ligand RSL1. Supernatants collected daily starting 3 dpi from these cell cultures were then examined for infectivity using TZM-bl cells and the results from 5 dpi are shown.

As another means to eliminate potential artifacts, we exploited the reversible nature of the RHGP technology. To eliminate clones that might have survived viral infection as a result of events unrelated to RHGP, HIV propagation was compared in the presence or absence of ligand RSL1 during HIV-1 re-challenge. Each of the RHGP-transduced clones demonstrated reversible resistance to HIV-1 infection (see a representative cell clone in Figure 6A). In the absence of exogenous ligand, we observed robust viral production that was comparable to parental controls.

To preclude that the act of the GSV integration into the MT4 genome might itself impart a nonspecific impact on HIV-1 replication, we tested naïve MT4 RHGP clones that had never previously been challenged with HIV-1. As a representative example, Clone H6 (in which the GSV integrates into the human SC6A3 gene) demonstrated no resistance to HIV-1, producing levels of HIV-1 production comparable to parental MT4 cells (Figure 6B). Likewise, HIV-1 infected H6 cells were completely depleted after infection (data not shown), thus confirming the specificity of the HIV-resistance demonstrated by the RHGP strategy.

### Identification of the host gene by genomic DNA cloning

**Figure 7:**
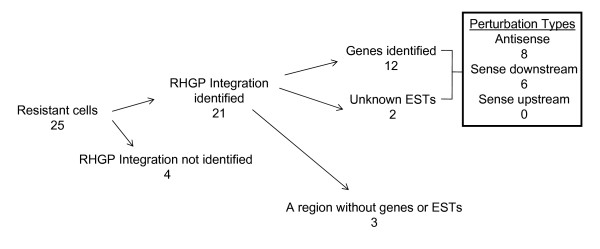
**An overview of HIV-1 resistant RHGP cell clones and subsequent discovery of integration locations**. Numbers of different integration occurrences are indicated blow each event.

To identify the targets perturbed by RHGP in the HIV-resistant MT4 cells, genomic DNA was isolated from the clones that demonstrated reversible resistance to HIV-1 (Figure 3, step 4). The 25 HIV-insensitive host cell clones with GSV integration sites yielded the identification of 21 cellular integration events (Figure 7). These GSV integrations targeted 12 previously-annotated genes and 2 non-annotated ESTs. Some clones were deemed progeny from a common parent since the GSV had integrated in the same genetic location with the same orientation. Three clones had RHGP insertions in a region without genes or ESTs. We were unable to isolate candidate genes from 4 cell clones due to partial loss of the Ori-CAT reporter.

The properties of these genes and ESTs are listed in Table 1. The site and orientation of integration offered by RHGP provided insight into the types of perturbations that allowed host cells to survive challenge with HIV-1. Specifically, the RHGP perturbations could be broadly divided into three groups: 1) "Antisense": Antisense integration events that facilitated gene expression disruption of one allele and antisense inhibition of gene expression from the other allele; 2) "Sense Downstream": Integration in a sense orientation, which would be predicted to facilitate production of a dominant-negative inhibitor of the endogenous gene product; and 3) "Sense-Upstream": Integration in a sense orientation upstream of the translation start site, which would be predicted to facilitate over-expression of the target gene. Of the 14 gene targets identified using RHGP, 8 targets represented "Antisense" knockdown of target expression. The other 6 of the targets represented "Sense-Downstream" events, likely representing over-expression of dominant-negative inhibitors of wild-type gene expression. No "Sense-Upstream" insertions were identified in the current study (Figure 7 and Table 1). Based on these predictions, all of the candidate genes are likely down-regulated by a GSV integration event. This allowed us to directly use siRNA knock down approach on naïve MT4 cells to recapitulate viral resistant phenotypes. Altogether, these findings suggest that RHGP-based interrogation of the host genome had identified both novel targets and/or ascribed novel functions to known genes.

**Table 1:** Summary of properties of identified HIV-1 resistant cellular genes

**Target genes**	**Main known function**	**Cellular localization**	**C'#**	**Perturbation**	**siRNA validation**
CASD1 CAS1 domain-containing protein 1 precursor	O-acetyl-transferase	Multi-pass membrane protein	7	Antisense intron 4 Down-regulation	Yes

HECW2 HECT, C2 and WW domain-containing protein 2	NEDD4-like E3 ubiquitin-protein ligase 2	Intracellular	2	Sense intron15 ATG(sc) exon 7 Down-regulation (DN)	Yes

ROBO1 roundabout protein homolog 1	axon guidance receptor and cell adhesion receptor	Type I membrane protein	3	Sense intron 3 ATG(sc) exon 3 Down-regulation (DN)	Yes

NLGN1 member of the neuroligin family	splice site-specific ligands for beta-neurexins and may be involved in the formation and remodeling of central nervous system synapses	Neuronal cell surface	3	Antisense intron 3 Down-regulation	Yes

DZIP3 (DAZ-interacting protein 3)	E3 Ubiquitin ligase proteins. Specifically bind RNA. Also called huRUL138 (RNA-binding ubiquitin ligase of 138 kDa)	Intracellular	3	Antisense intron 1 Down-regulation	Yes

CAMSAP1L1 calmodulin regulated spectrin-associated protein	may be involved in spectrin's function as a cytoskeletal protein providing a scaffolding and maintenance of plasma membrane	Membrane	1	Antisense intron 2 Down-regulation	Yes

GSTCD glutathione S-transferase (GST), C-terminal domain	structural domain of GST, which conjugates reduced glutathione to a variety of targets to facilitate detoxification of the targets	Intracellular	4	Antisense intron 5 Down-regulation	Yes

CPSF1 Cleavage and polyadenylation specificity factor	It recognizes the AAUAAA signal in the pre-mRNA and facilitates both RNA cleavage and polyA synthesis	Nucleus	8	Antisense intron 2 Down-regulation	Yes

GDAP2 ganglioside induced differentiation associated protein 2	a signal transduction pathway during neuronal development	Intracellular	1	Sense last exon Down-regulation (DN)	Yes

TNRC6A trinucleotide repeat containing 6 protein	post-transcriptional gene silencing through the RNAi and microRNA pathways.	Cytoplasmic bodies	16	Sense intron 4 ATG(sc) exon 1 Down-regulation (DN)	Yes

TTC21B tetratricopeptide repeat domain 21B	may negatively modulate SHH (Sonic Hedgehog) signal transduction and may play a role in retrograde intraflagellar transport in cilia	Intracellular	2	Antisense intron 4 Down-regulation	Yes

ATP8A1 aminophospholipid transporter (APLT), Class I, type 8A, member 1	transport of aminophospholipids from the outer to the inner leaflet of various membranes and the maintenance of asymmetric distribution of phospholipids, mainly in secretory vesicles	Membrane	4	Sense intron 4 ATG(sc) exon 1 Down-regulation (DN)	Yes

Unknown ESTs	BE066906, AW817767, EB388641	unknown	3	Sense	n.d.
	
	AW300614	unknown	2	Antisense	n.d.

Symbols, gene families and the currently main known functions are shown for each identified gene. C# indicates the chromosome number of each target gene locates. The disruption effects upon GSV integration (orientation and integrated sites in host genes) are also listed. In order to determine the possible effects of sense integration on host gene expression, locations of the translation start codon (ATG(sc)) are also indicated. Down-regulation of gene expression is expected for the targeted genes through anti-sense inhibition. Dominant Negative (DN) effects are also anticipated from the targeted genes with over-expression of a truncated host protein when GSV inserts downstream of the start codon ATG in the sense orientation. GenBank Access numbers and the disruption effects of GSV integration are also shown for the two unknown ESTs. "n.d." stands for "not done".

### Validation of target genes using naïve cells

The studies above demonstrated that RHGP could identify novel host targets that conferred resistance to HIV-1 infection. We then sought to verify these candidates using an independent experimental system to exclude outcomes that might arise as spontaneous mutation or unanticipated artifacts of the RHGP technology. Thus, duplex siRNAs targeting these candidates were obtained. Each siRNA preparation contained a pool of 4 individual siRNAs, all of which selectively target the gene of interest. Non-targeting siRNAs provided a matched control for the transfection and a reference standard. siRNA constructs specific for viral *Tat *and a cellular target, Rab6A, provided positive controls based on recent reports that these siRNA were able to efficiently inhibit HIV-1 infection [17].

**Figure 8:**
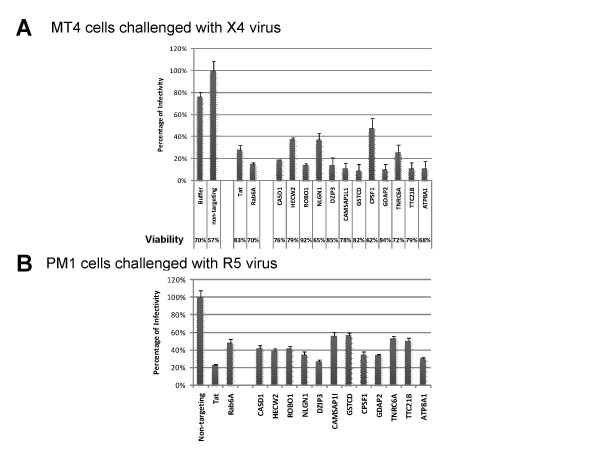
**Validation of candidate genes providing HIV-1 resistance via siRNA targeting of naïve T lymphocytes**. Naïve T cells were challenged one day after electroporation with the siRNAs specific for the human targets listed in Table 1. Production of the progeny virus 48 h post-infection were quantified using TZM-bl cells. Percentages of virus production from each siRNA treatment against the non-targeting siRNA control sample are shown. Note that siRNA against Tat and Rab6A provided positive controls for established HIV-1 siRNA targets. **(A) **Data from siRNA transfected MT4 cells followed by X4 tropic HIV-1_NL4-3 _infection. Percentages of viable cells after siRNA transfection and viral infection (2 dpi) are shown in the bottom. **(B) **Results from siRNA transfected PM1 cells followed by infection with the R5 tropic HIV-1_Mei _virus.

The siRNAs were transfected into naïve MT4 cells *via *electroporation one day prior to challenge with HIV-1_NL4-3_. Culture supernatants were harvested two days after infection and the number of infectious virions was measured using TZM-bl cell-based readouts. As indicated in Figure 8A, duplex siRNAs against the 12 target genes reduced HIV-1 virus production by 50-90%, which was comparable to the inhibition observed in the positive controls (Tat and Rab6A). As a control, we also evaluated the overall viability of the MT4 host cells, which allowed us to exclude cytotoxic effects that have arisen from siRNA treatment and thus decreased viral release as a result of a general decrease in cell viability (rather than a specific impact on the viral life cycle). Despite the inhibition of HIV-1 release, the viability of siRNA-treated samples was comparable in all samples. These results confirmed that these genes identified by RHGP are important in viral replication and validated the application of RHGP to identify novel host-based targets.

An important goal of our present studies was to identify targets that are broadly applicable to HIV-1 infection. We also sought to confirm that targets identified using RHGP would not be unique to any particular cell system. To address both issues, we asked if the host gene candidates that rendered MT4 cells insensitive to challenge by HIV-1_NL4-3 _(a CXCR4-tropic virus) would similarly allow a different cell system to become insensitive to challenge by a CCR5 tropic HIV-1 virus. For this, the same siRNA approach as used with MT4 cells was used to target relative molecules in PM1 T cells. PM1 was selected since it expresses both CXCR4 and CCR5 co-receptors and thus can provide a model for both R5 and X4-tropic viruses [11]. Similar to our findings with CXCR4-tropic viruses, targeting in PM1 cells demonstrated that this same set of 12 siRNAs was able to inhibit viral replication of the R5-tropic HIV-1_ME1_[14]. Viral production of HIV-1_ME1 _strain was significantly inhibited in the cells treated with specific siRNA targeting each of these 12 gene targets (Figure 8B). These results confirmed our findings that the targets identified using RHGP are important for the replication of both X4 and R5 tropic HIV-1 viruses.

**Figure 9:**
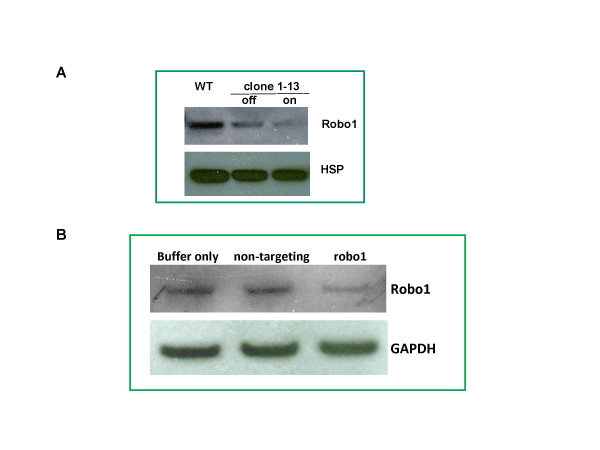
**Confirmation of target gene expression using Robo1 as an example**. **(A)**. Loss of Robo1 expression in the RHGP clone whose robo1 gene was perturbed by RHGP in the presence of RSL1 (on) and reemergence of expression in the absence of RSL1 (off). Cell lysates of WT MT4 cells and RHGP clone were resolved in an SDS-PAGE gel. After transfer to a membrane, the blot was first probed by anti-Robo1 (A301-265A, Bethyl Laboratories) and then anti-HSP90 (heat shock protein) after stripping, each followed by 2^nd ^HRP-conjugated anti-rabbit Ab. **(B)**. Knockdown expression of Robo1 in MT4 cells treated with siRNA against robo1. Cell lysates from samples treated with different siRNA as indicated were processed into western blot as described above. Herein GAPDH protein probed by an anti-GAPDH was used as a loading control.

In the course of validating targets identified using RHGP, we identified novel mechanistic information about certain target functions. For example, in clone #1-13, the integration of the GSV occurred in a "sense downstream" after start codon of the human Robo1 gene. This integration was predicted to result in the production of a truncated form of Robo1 (table 1). Western blot analysis with Robo1 specific antibodies indicated that expression of wild type Robo1 (~220 kDa) in clone #1-13 was down regulated after GSV integration (Figure 9A). Other immunoglobulin superfamily members require multimerization [19,20] and improperly folded multimers are likely to be efficiently degraded. Thus, we reasoned that the truncated molecule might favor degradation of endogenous Robo1. When the RHGP promoter turned "off" upon withdrawal of ligand RSL1, the truncated protein was no longer produced and normal levels of Robo1 expression reemerged (Figure 9A). Likewise, viral replication increased upon removal of RSL1 (Figure 6A), which directly related to the restoration of wild-type Robo1 protein.

To validate the targets identified using RHGP, we sought to reproduce the perturbation in a "naïve" cell that has not been modified by the GSV. To verify that the siRNA targeting Robo1 in naïve T cells significantly reduced viral production during HIV-1 infection (Figure 8), we next examined whether Robo1 expression was successfully knocked down upon siRNA treatment using western blot. Indeed, reduced amounts of Robo1 were found in the siRNA treated cells (Figure 9B).

### Resistance of RHGP cell clones to drug-resistant HIV-1

**Figure 10:**
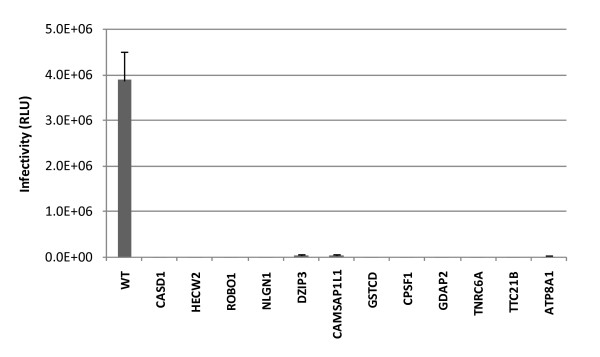
**Failure to produce virus by the HIV-1 resistant RHGP cell clones upon challenge by a protease inhibitor resistant HIV-1 mutant**. Production of progeny virus were examined on supernatants by TZM-bl cells after cells from parental MT4-R1 and resistant cell clones were challenged with the protease inhibitor resistant HIV-1 mutant (L10R/M46I/L63P/V82T/I84V). Results from 4 dpi are shown.

Although the results with wild-type HIV-1 were encouraging, we considered that a large unmet need for therapeutics is the application of new targets to viral variants that are resistant to conventional medicines. Therefore, we performed studies with an HIV-1 variant (L10R/M46I/L63P/V82T/I84V) with established resistance to protease inhibitors[15]. The RHGP-transduced clones selected after wild type HIV-1_NL4-3 _challenge also survived challenge in the face of the protease-resistant variant and failed to produce viruses after challenge. This outcome was not unique to host cell survival as infectivity assays as well as p24 ELISA confirmed the defective infection by mutant HIV-1 in the resistant cells (Figure 10 and data not shown). Together these results confirmed the cell clones we obtained are resistant to infection by both wild type and drug resistant HIV-1 variants and further indicated that therapeutics based on the identified gene targets have the broad spectrum potential against replication of HIV mutants resistant to current anti-viral drugs.

## Discussion

In our present study, we applied RHGP technology to conduct a genome-wide screen for host factors required for HIV-1 virus infection and identified novel host-based targets that render cells resistant to an otherwise lethal challenge with HIV-1 virus. In addition, we ascribed novel anti-HIV-1 functions to previously-known genes as well as non-annotated ESTs. These targets were validated first using an inducible promoter incorporated within the RHGP vector to reverse the phenotype and then in naïve cells using the conventional siRNA approach. We further found that the resultant targets were broadly applicable to different HIV variants, including CCR5 and CXCR4-tropic viruses. We further showed that cell clones with the gene targets disrupted by RHGP were resistant to viral challenge by a drug resistant HIV-1 mutant.

An independent study from our group recently identified host targets that allow host cells to survive in the face of an otherwise lethal infection with influenza virus[10]. That study, as well as the work herein, employed a lentiviral system to overcome the prior limitation of low GSV production, which had been a problem associated with Moloney murine leukemia virus (MMLV)-based strategies [9]. These improvements allowed us to sample the entire host genome and identify 12 gene targets from the 10^5 integration events invoked by the improved GSV. Consistent with prediction that a lentiviral vector favors single site insertion into sites of active gene transcription [21], all integration occurred in regions with active gene expression.

Based on MMLV-derived vectors which randomly integrate into the host chromosome, insertional mutagenesis was described as a high-throughput forward genetics approach to inactivate and thus discover cellular genes. Using these vectors cellular genes were identified that are required for replication of HIV-1 and other viruses, but not for host cell survival [22,23]. With a built-in inducible promoter in GSV to drive transcript production from a host gene, our RHGP can also generate activation, often over-expression, of genes in mammalian cells, depending on the location and direction of GSV insertion relative to the candidate gene. Although not observed in our current study, over-expression of a subset of targets including the B-cell CLL/lymphoma 2 (BCL2) allowed MDCK cells to survive influenza infection during discovery of host genes against influenza virus infection[10]. Since target expression is under control of the inducible promoter, the causal relationship between the phenotype (resistance to HIV-1 infection) and the perturbed gene can be confirmed by withdrawal of the inducer. By validating these targets within the same experiment, this feature markedly increases the efficiency of discovery of therapeutic candidates. Indeed, all the targets from resistant clones were successfully confirmed in the subsequent siRNA studies with naïve cells. Instead of transient knock down effects generated by conventional approaches (e.g., siRNA), the RHGP phenotype is highly stable, which can allow mechanistic studies to continuously characterize the roles of these perturbed genes in HIV-1 replication.

Increasing evidence suggests that the concept of host-oriented therapeutics may be particularly useful for identifying improved opportunities to combat HIV/AIDS. To identify relevant host targets, recent siRNA or shRNA based genome-wide studies have successfully identified host targets associated with HIV-1 infection [17,24-26]. Unfortunately, siRNA is intrinsically limited by the need for strong and stable over-expression of the siRNA. Moreover, the outcomes of some siRNA findings have been clouded by recent questions of whether the siRNAs might non-specifically alter host defense mechanisms [27], which could be particularly problematic for applications of siRNA technology to therapies against viral diseases. Any siRNA screen is likely to generate false positive and false negative data due to the potential "off-target effects" along with variability both in siRNA efficacy and protein half-lives. This variability will ultimately lead to different levels of protein knockdown. We believe RHGP could provide an alternative since it is not limited by these same constraints.

Notably, the genes indentified herein are not included in the list discovered by the three recent siRNA-based HIV-1 studies [17,24,25]. We postulate this discrepancy may reflect that the different cell systems, viruses and approaches were used during screening coupled with contrasting experimental designs. Consistent with this postulation, there was very limited overlap between the HIV dependency factors identified in these investigations[28]. These earlier studies did not utilize CD4^+ ^T lymphocytes, a natural cell target for HIV infection. To achieve efficient siRNA delivery and investigate viral replication in those studies, adherent HeLa -derived cells were reconstructed to express CD4 and CXCR4 (such as TZM-bl cells used in the Brass et al screen [17] or P4/R5 cells in the Zhou et al study [25]). In a third investigation, a mutant HIV-1 virus pseudotyped with VSV-G was used to allow virus entry [24]. This raises a question as to whether such differences might alter the mechanism of viral replication. Consistent with this, defective viral budding was observed in TZM-bl cells (our unpublished data). Using CD4^+ ^T lymphocytes and wild type HIV-1 virus, we were able to interrogate very natural interactions between the entire repertoire of host factors and viral proteins during complete cycles of viral replication. We also noted that while we sampled the whole genome, a relatively low number of host genes (<20) were identified in our study. This subset of candidates might have resulted from the fact that our strategy of cloning surviving cells might have precluded targets that impact cell growth rates or viability. Likewise, the use of cell models and laboratory adapted viruses might have limited or biased the repertoire of host targets identified using RHGP. Thus, future studies should seek to adapt this technology using virus isolates obtained from primary cells.

A central tenet of the RHGP technology is that it is not biased by prior knowledge of the target. Consistent with this, two (of 14) that render host cells resistant to HIV infection were ESTs that had not yet been annotated (Table 1). Analysis of target function via the PANTHER Classification System [29], indicated little (CASD1, GDAP2, TNRC6A, and TTC21B,) or no (CAMSAP1L1 and GSTCD) knowledge of target function. The function for four targets have been ascribed (ROBO1, NLGN1, CPSF1, ATP8A1), but none of them have been linked with viral infection. The biological processes that these targets are involved in diversely include mRNA polyadenylation (CPSF1), cation transport (ATP8A1) and cell adhesion mediated signaling (ROBO1 and NLGN1).

HECW2 and DZIP3 are members of the E3 ubiquitin ligase family. This finding is intriguing since the ubiquitin ligase pathway has been shown to be necessary for the budding and release of HIV-1 and other viruses. For example, the TSG101 host protein is a ubiquitin-like ligase that interacts with HIV-1-encoded p6 Gag and is "hijacked" to facilitate viral egress from the cell surface [6]. Likewise, Nedd4 is another ubiquitin ligase that regulates viral budding and release including HIV-1 [30,31]. It is notable that HECW2 shares considerable homology with Nedd4 [32]. It is thus tempting to postulate that HECW2 and DZIP3 may be also important for HIV-1 maturation and egress.

Robo1 is also essential for HIV replication. Viral production was markedly inhibited in both RHGP promoter "on" cells and siRNA treated naïve cells where knockdown of Robo1 expression were observed (Figure 8 and 9). This consistency was demonstrated again in RHGP cells in the subsequent reversibility assay when inducible promoter turned off and where levels of viral replication increased with the reemergence of Robo1 expression (Figure 9A). Robo1 is a type I transmembrane protein with an extracellular N-terminus comprising of 5 immunoglobulin and 3 fibronectin domains [20]. It was originally identified as axon guidance receptor during neuronal development and was recently shown to regulate T cell chemotaxis [33,34].

Our work thereby provides potential insights into new opportunities for host-directed therapeutics. Specifically, novel technologies like RHGP provide an opportunity to identify and prioritize host molecules that might provide safe and effective targets for drug intervention. In light of the increasing evidence that different virus types can share essential host pathways during their replication cycles, the therapeutics developed from genes identified in this study might also have broad application to other viruses as well.

## Conclusion

Using RHGP, we have successfully identified novel host cell targets that are essential for HIV-1 replication but which can be safely targeted to preclude damage to normal cells. These targets emphasize safety while effectively blocking viral propagation. Most current HIV drugs target the HIV virus and therefore are vulnerable to the development of drug resistance through viral mutation. In contrast, therapeutics based on these newly identified human host targets will prevent HIV virus from using the host's cellular mechanism for its life cycle and are insensitive to drug resistance. Moreover, by targeting cellular pathways shared by HIV variants and even viruses other than HIV, these therapies have potentially broad spectrum anti-viral activities.

## Competing interests

All authors are inventors on a patent application, which describes applications of methods and results presented herein. The intellectual property rights are owned by Functional Genetics, Inc.

## Authors' contributions

HM participated in the conception, design, coordination and conductions of the study; HC carried out most of experiments in the study; ZF examined amounts of HIV p24; SC, HU, JVD, and MK participated in cloning of target genes; HU performed western blot analysis; WL and MK designed and constructed RHGP vectors; MSK and MG helped the conception and design of this study. MSK, together with HM, wrote the manuscript. All authors have read and approved the manuscript.

## References

[B1] Grant RM, Hecht FM, Warmerdam M, Liu L, Liegler T, Petropoulos CJ, Hellmann NS, Chesney M, Busch MP, Kahn JO (2002). Time trends in primary HIV-1 drug resistance among recently infected persons. JAMA.

[B2] Richman DD, Morton SC, Wrin T, Hellmann N, Berry S, Shapiro MF, Bozzette SA (2004). The prevalence of antiretroviral drug resistance in the United States. AIDS.

[B3] Liu R, Paxton WA, Choe S, Ceradini D, Martin SR, Horuk R, MacDonald ME, Stuhlmann H, Koup RA, Landau NR (1996). Homozygous defect in HIV-1 coreceptor accounts for resistance of some multiply-exposed individuals to HIV-1 infection. Cell.

[B4] Samson M, Libert F, Doranz BJ, Rucker J, Liesnard C, Farber CM, Saragosti S, Lapoumeroulie C, Cognaux J, Forceille C (1996). Resistance to HIV-1 infection in caucasian individuals bearing mutant alleles of the CCR-5 chemokine receptor gene. Nature.

[B5] Moore JP (1997). Coreceptors: implications for HIV pathogenesis and therapy. Science.

[B6] Garrus JE, von Schwedler UK, Pornillos OW, Morham SG, Zavitz KH, Wang HE, Wettstein DA, Stray KM, Cote M, Rich RL (2001). Tsg101 and the vacuolar protein sorting pathway are essential for HIV-1 budding. Cell.

[B7] Wheeler J, McHale M, Jackson V, Penny M (2007). Assessing theoretical risk and benefit suggested by genetic association studies of CCR5: experience in a drug development programme for maraviroc. Antiviral Therapy.

[B8] Reeves JD, Piefer AJ (2005). Emerging drug targets for antiretroviral therapy. Drugs.

[B9] Li L, Cohen SN (1996). Tsg101: a novel tumor susceptibility gene isolated by controlled homozygous functional knockout of allelic loci in mammalian cells. Cell.

[B10] Sui B, Bamba D, Weng K, Ung H, Chang S, Van Dyke J, Goldblatt M, Duan R, Kinch MS, Li WB (2009). The use of Random Homozygous Gene Perturbation to identify novel host-oriented targets for influenza. Virology.

[B11] Lusso P, Cocchi F, Balotta C, Markham PD, Louie A, Farci P, Pal R, Gallo RC, Reitz MS (1995). Growth of macrophage-tropic and primary human immunodeficiency virus type 1 (HIV-1) isolates in a unique CD4+ T-cell clone (PM1): failure to downregulate CD4 and to interfere with cell-line-tropic HIV-1. J Virol.

[B12] Wei X, Decker JM, Liu H, Zhang Z, Arani RB, Kilby JM, Saag MS, Wu X, Shaw GM, Kappes JC (2002). Emergence of resistant human immunodeficiency virus type 1 in patients receiving fusion inhibitor (T-20) monotherapy. Antimicrob Agents Chemother.

[B13] Adachi A, Gendelman HE, Koenig S, Folks T, Willey R, Rabson A, Martin MA (1986). Production of acquired immunodeficiency syndrome-associated retrovirus in human and nonhuman cells transfected with an infectious molecular clone. J Virol.

[B14] Chen M, Singh MK, Balachandran R, Gupta P (1997). Isolation and characterization of two divergent infectious molecular clones of HIV type 1 longitudinally obtained from a seropositive patient by a progressive amplification procedure. AIDS Res Hum Retroviruses.

[B15] Condra JH, Schleif WA, Blahy OM, Gabryelski LJ, Graham DJ, Quintero JC, Rhodes A, Robbins HL, Roth E, Shivaprakash M (1995). In vivo emergence of HIV-1 variants resistant to multiple protease inhibitors. Nature.

[B16] Lu Q, Wei WS, Kowalski PE, Chang ACY, Cohen SN (2004). EST-based genome-wide gene inactivation identifies ARAP3 as a host protein affecting cellular susceptibility to anthrax toxin. Proceedings of the National Academy of Sciences of the United States of America.

[B17] Brass AL, Dykxhoorn DM, Benita Y, Yan N, Engelman A, Xavier RJ, Lieberman J, Elledge SJ (2008). Identification of host proteins required for HIV infection through a functional genomic screen. Science.

[B18] Gupta PK, Varshney RK (2004). Cereal genomics: An overview. Cereal Genomics.

[B19] Brummendorf T, Lemmon V (2001). Immunoglobulin superfamily receptors: cis-interactions, intracellular adapters and alternative splicing regulate adhesion. Curr Opin Cell Biol.

[B20] Hohenester E (2008). Structural insight into Slit-Robo signalling. Biochem Soc Trans.

[B21] Mitchell RS, Beitzel BF, Schroder ARW, Shinn P, Chen HM, Berry CC, Ecker JR, Bushman FD (2004). Retroviral DNA integration: ASLV, HIV, and MLV show distinct target site preferences. Plos Biology.

[B22] Murray JL, Mavrakis M, McDonald NJ, Yilla M, Sheng J, Bellini WJ, Zhao L, Le Doux JM, Shaw MW, Luo CC (2005). Rab9 GTPase is required for replication of human immunodeficiency virus type 1, filoviruses, and measles virus. J Virol.

[B23] Bruce JW, Ahlquist P, Young JA (2008). The host cell sulfonation pathway contributes to retroviral infection at a step coincident with provirus establishment. PLoS Pathog.

[B24] Konig R, Zhou YY, Elleder D, Diamond TL, Bonamy GMC, Irelan JT, Chiang CY, Tu BP, De Jesus PD, Lilley CE (2008). Global analysis of host-pathogen interactions that regulate early-stage HIV-1 replication. Cell.

[B25] Zhou H, Xu M, Huang Q, Gates AT, Zhang XD, Castle JC, Stec E, Ferrer M, Strulovici B, Hazuda DJ, Espeseth AS (2008). Genome-scale RNAi screen for host factors required for HIV replication. Cell Host Microbe.

[B26] Yeung ML, Houzet L, Yedavalli VS, Jeang KT (2009). A genome-wide short hairpin RNA screening of jurkat T-cells for human proteins contributing to productive HIV-1 replication. J Biol Chem.

[B27] Kleinman ME, Yamada K, Takeda A, Chandrasekaran V, Nozaki M, Baffi JZ, Albuquerque RJC, Yamasaki S, Itaya M, Pan YZ (2008). Sequence- and target-independent angiogenesis suppression by siRNA via TLR3. Nature.

[B28] Bushman FD, Malani N, Fernandes J, D'Orso I, Cagney G, Diamond TL, Zhou H, Hazuda DJ, Espeseth AS, Konig R (2009). Host cell factors in HIV replication: meta-analysis of genome-wide studies. PLoS Pathog.

[B29] Mi HY, Lazareva-Ulitsky B, Loo R, Kejariwal A, Vandergriff J, Rabkin S, Guo N, Muruganujan A, Doremieux O, Campbell MJ (2005). The PANTHER database of protein families, subfamilies, functions and pathways. Nucleic Acids Research.

[B30] Bieniasz PD (2006). Late budding domains and host proteins in enveloped virus release. Virology.

[B31] Chen BJ, Lamb RA (2008). Mechanisms for enveloped virus budding: can some viruses do without an ESCRT?. Virology.

[B32] Miyazaki K, Ozaki T, Kato C, Hanamoto T, Fujita T, Irino S, Watanabe K, Nakagawa T, Nakagawara A (2003). A novel HECT-type E3 ubiquitin ligase, NEDL2, stabilizes p73 and enhances its transcriptional activity. Biochem Biophys Res Commun.

[B33] Prasad A, Qamri Z, Wu J, Ganju RK (2007). Slit-2/Robo-1 modulates the CXCL12/CXCR4-induced chemotaxis of T cells. J Leukoc Biol.

[B34] Wu JY, Feng L, Park HT, Havlioglu N, Wen L, Tang H, Bacon KB, Jiang Z, Zhang X, Rao Y (2001). The neuronal repellent Slit inhibits leukocyte chemotaxis induced by chemotactic factors. Nature.

